# Diagnostic Value of Six Tumor Markers for Malignant Pleural Effusion in 1,230 Patients: A Single-Center Retrospective Study

**DOI:** 10.3389/pore.2022.1610280

**Published:** 2022-04-20

**Authors:** Xin Fan, Yanqing Liu, Zhigang Liang, Shanshan Wang, Jing Yang, Aihua Wu

**Affiliations:** ^1^ Department of Dermatology, Ningbo First Hospital, Ningbo, China; ^2^ Department of Laboratory Medicine, Ningbo First Hospital, Ningbo, China; ^3^ Department of Thoracic Surgery, Ningbo First Hospital, Ningbo, China; ^4^ Department of Respiratory and Critical Care, Ningbo First Hospital, Ningbo, China

**Keywords:** diagnostic performance, malignant pleural effusion, tumor markers, carcinoembryonic antigen, cytokeratin 19 fragment, area under the curve

## Abstract

**Background:** The diagnostic value of tumor markers in pleural effusion (PE) and serum for malignant pleural effusion (MPE) is still in debate. This study aimed to evaluate the diagnostic value of six tumor markers in PE, serum, and the corresponding PE/serum (PE/S) ratio in distinguishing MPE from benign pleural effusion (BPE).

**Methods:** A total of 1,230 patients with PE (452 MPEs and 778 BPEs) were retrospectively included in the study. PE and serum levels of carcinoembryonic antigen (CEA), carbohydrate antigen 15-3 (CA15-3), carbohydrate antigen 125 (CA125), carbohydrate antigen 19-9 (CA19-9), cytokeratin 19 fragment (CYFRA 21-1), and neuron-specific enolase (NSE) were measured. The area under the curve (AUC) was used to assess the single and combined diagnostic values of the six tumor markers for MPE.

**Results:** The levels of the six tumor markers in PE, serum, and PE/S were significantly higher in MPE than that in BPE, except for serum CA125. PE CEA showed the highest AUC [0.890 (0.871–0.907)] at a cut-off value of 3.7 ng/ml compared to any single tumor marker using receiver operating characteristic (ROC) analysis. The specificity, sensitivity, positive predictive value (PPV), negative predictive value (NPV), positive likelihood ratio (PLR), and negative likelihood ratio (NLR) of PE CEA were 74.1%, 95.5%, 90.5%, 86.4%, 16.47, and 0.27, respectively. The combination of PE CEA and serum CYFRA21-1 showed the best diagnostic performance with an AUC of 0.934 (sensitivity, 79.9%; specificity, 95.7%, PPV, 90.5; PLR, 17.35) among all two or three combinations. Besides, serum CYFRA21-1 was the best diagnostic tumor marker in distinguishing cytology-negative MPE from BPE at a cut-off value of 3.0 ng/ml.

**Conclusion:** PE CEA was the best diagnostic tumor marker in distinguishing MPE from BPE. Serum CYFRA21-1 was the best diagnostic tumor marker in distinguishing cytology-negative MPE from BPE. The combination of PE CEA and serum CYFRA21-1 could increase the diagnostic performance in distinguishing MPE from BPE and cytology-negative MPE from BPE.

## Introduction

Pleural effusion (PE) is a clinically common complication in patients with physical traumata or systemic disorders, such as cancer, inflammation, and infection [1, 2]. A malignant tumor is one of the main causes leading to PE, and more than 90% of malignant pleural effusion (MPE) is due to metastatic disease, which might endanger the patient’s survival [3]. Therefore, elucidating the etiologies of PE is critically important for involving treatment options and prognoses of PE patients, especially to differentiate MPE from benign pleural effusion (BPE). The negative rate of conventional PE cytology in malignant PEs can be as high as 40%, and even higher in PEs of squamous-cell lung carcinomas and mesotheliomas [4, 5]. The cytological examination is influenced not only by tumor types, but the number of analyzed specimens, cytologist’s experience, and the volume of pleural fluid processed [1, 3]. In addition, under some circumstances, non-specific inflammatory PE derived from lung tumor development, lymphatic obstruction, and/or immune-mediated inflammation might account for the failure of a cytology examination. Although thoracoscopy and/or thoracotomy presented high diagnostic sensitivity for MPE, the safety and need for a confirmatory pleural biopsy in patients with suspected MPE or cytology-negative MPE in studies vary widely [6, 7].

On this occasion, tumor markers have been reported to aid the diagnosis of malignancy, such as carcinoembryonic antigen (CEA), carbohydrate antigen 15-3 (CA15-3), carbohydrate antigen 125 (CA125), and cytokeratin 19 fragment (CYFRA 21-1) [8]. Besides, tumor markers in PE have been considered to be less invasive for differentiating MPE from BPE. Although those tumor markers have been extensively assessed for distinguishing between MPE and BPE in numerous studies, the inconsistent cut-offs, sensitivities, and specificities of those tumor markers for definitive diagnosis of MPE have raised controversies [8, 9].

Therefore, the present study aimed to evaluate six tumor markers (CEA, CA15-3, CA125, CA19-9, CYFRA21-1, and NSE) in PE, serum, and the corresponding PE/serum (PE/S) ratio in distinguishing MPE from BPE either singly or in combination. We also assessed the diagnostic value of the aforementioned six tumor markers in distinguishing cytology-negative MPE from BPE either singly or in combination.

## Materials and Methods

### Patients

Pleural effusion was examined by thorough anamnesis, physical examination (lung percussion and auscultation), X-ray, or chest CT, and was confirmed by thoracentesis. A total of 1,230 patients with PE admitted to the Department of Thoracic Surgery and Respiratory and Critical Care of Ningbo First Hospital from 1 January 2014 to 1 March 2021 were enrolled and analyzed retrospectively. The diagnosis of MPE or BPE was made based on the combination of cytology, thoracoscopy, imagological examination, and at least a 6-month follow-up. The cytology examination was performed on stained slides of fresh PE samples by two pathologists. Based on cytological results, MPE was classified into three categories, namely positive cytology (*n* = 262), suspected cytology (*n* = 60), and negative cytology (*n* = 130). The causes of MPE (*n* = 452) and BPE (*n* = 778) in the 1,230 patients are shown in Table 1. As shown in Table 1, lung cancer (78.8%), breast cancer (3.3%), and gastric cancer (2.6%) were the leading causes of MPE. Tuberculosis (49.5%), parapneumonic (19.4%), and congestive heart failure (10%) were the leading causes of BPE. Besides, PEs from patients with a history of definite malignant tumors was also considered to be malignant if other diseases were excluded. The exclusion criteria were as follows: 1) younger than 18 years old; 2) pregnant women; and 3) incomplete data. Besides, patients initially diagnosed with BPE were excluded if they developed any tumor during follow-up periods. Mesothelioma and hematological malignancy were also excluded for not frequently elevated CEA when PE was caused these malignancies. The clinicopathological characteristics, including age, gender, smoking history, level of six tumor markers (CEA, CA15-3, CA125, CA19-9, CYFRA 21-1, and NSE) in PE and serum, and cytological data were obtained from the electronic medical record system for patients. The present study was performed in accordance with the Declaration of Helsinki and approved by the Ethics Committee of Ningbo First Hospital (No. 2021RS133).

**TABLE 1:** Etiology of PEs of 1,230 patients.

Causes of PEs	Number of cases (%)
MPE	452
Lung cancer	356 (78.8%)
Breast cancer	15 (3.3%)
Gastric cancer	12 (2.6%)
Ovarian cancer	9 (2.0%)
Liver cancer	8 (1.8%)
Esophageal cancer	6 (1.3%)
Colorectal cancer	6 (1.3%)
Others^a^	8 (1.8%)
Unknown origin	32 (7.1%)
BPE	778
Tuberculosis	385 (49.5%)
Parapneumonic	151 (19.4%)
Congestive heart failure	78 (10.0%)
Empyema	58 (7.5%)
Parasitic	24 (3.1%)
Postsurgery	8 (1.0%)
Miscellaneous	40 (5.1%)
Non-neoplastic unknown etiology	34 (4.4%)

PE, pleural effusion; MPE, malignant pleural effusion; BPE, benign pleural effusion.

a Others included bladder cancer (*n* = 1), thyroid carcinoma (*n* = 1), renal-cell carcinoma (*n* = 1), prostate carcinoma (*n* = 2), nasopharyngeal carcinoma (*n* = 1), laryngeal cancer (*n* = 1), and synovial sarcoma (*n* = 1).

### PE and Serum Tumor Markers Analysis

PE samples were collected from all patients by standard thoracentesis within 24 h of admission. Fasting peripheral blood samples (4.0 ml) were drawn from all patients before treatment. PE and blood samples were transported to the Department of Clinical Laboratory Medicine within 1 h. CEA, CA15-3, CA125, and CA19-9 were detected by a chemiluminescence method (Cobas e602, Roche Diagnostics, Germany), and CYFRA21-1 and NSE were detected by an electrochemical luminescence method (Cobas e602, Roche Diagnostics, Germany) with commercial assay kits according to the manufacturer’s instructions. The reference interval of the aforementioned tumor markers in serum was recommended as follows: 5 ng/ml for CEA, 14 U/ml for CA15-3, 35 U/ml for CA125, 25.0 ng/ml for CA19-9, 3.3 ng/ml for CYFRA 21-1, and 16.3 ng/ml for NSE.

### Statistical Analysis

All statistical analyses were performed using MedCalc version 18.0 (MedCalc Software Ltd., Ostend, Belgium) and R Studio software version 4.0.5 (http://www.r-project.org) with the OptimalCutpoints package. A two-tailed *p* < 0.05 was considered to be significantly different.

Categorical variables were determined by the Chi-squared (*χ* ^2^) test. Continuous variables were presented as mean ± standard deviation (SD). Continuous variables were determined by the nonparametric Mann-Whitney U test. The optimal cut-off value, area under the curve (AUC), sensitivity, specificity, positive predictive value (PPV), negative predictive value (NPV), positive likelihood ratio (PLR), and negative likelihood ratio (NLR) of each tumor marker and the corresponding PE/serum ratio were calculated by R Studio software. Besides, the better tumor markers with good diagnostic value were defined as an AUC greater than 0.75. The receiver operating characteristic (ROC) curves were calculated by MedCalc software.

## Results

### The Levels of Six Tumor Markers and Their Corresponding PE/S Ratios in Total MPE, Cytology-Negative MPE, and BPE Patients

The basic characteristics and six tumor marker levels in PE, serum, and PE/S among total MPE, cytology-negative MPE, and BPE are presented in Table 2. Among 1,230 patients, 452 patients had MPE (262 men, 190 women) and 778 had BPE (519 men, 259 women). Besides, 130 patients with MPE were cytology-negative. The average age of patients who had total MPE and cytology-negative MPE was older than that in BPE patients (67.8 years and 67.3 years versus 56.1 years, *p* < 0.001, Table 2). The gender between total MPE and BPE was statistically different (*p* = 0.003, Table 2), while that between cytology-negative MPE and BPE was not (*p* = 0.688, Table 2). No statistical difference was observed in smoking status between the three groups. The levels of CEA, CA15-3, CA125, CA19-9, CYFRA21-1, and NSE in PE, serum, and PE/S were all significantly higher in MPE patients than those in BPE patients except for PE/S CYFRA21-1 (*p* = 0.120, Table 2). However, the levels of CEA, CA19-9, and CYFRA21-1 in PE, serum, and PE/S were all significantly different between cytology-negative MPE and BPE (Table 2).

**TABLE 2:** Baseline characteristics, tumor markers levels in PE, serum and PE/S ratio among total MPE, cytology-negative MPE, and BPE.

Variables	BPE (*n* = 778)	Total MPE (*n* = 452)	Cytology-negative MPE (*n* = 130)	*p* ^a^ Value	*p* ^b^ Value
Age (years)	56.1 ± 20.7	67.8 ± 13.0	67.3 ± 12.8	<0.001	<0.001
Gender (n, %)					
Male	519 (66.7%)	262 (58.0%)	84 (64.6%)	0.003	0.688
Female	259 (33.3%)	190 (42.0%)	46 (35.4%)		
Smoking status (n, %)					
Non-smoker	494 (63.5%)	278 (61.5%)	77 (59.2%)	0.501	0.378
C/F smoker	284 (36.5%)	174 (38.5%)	53 (40.8%)		
PE CEA (ng/ml)	1.7 ± 5.6	280.9 ± 395.9	81.0 ± 227.0	<0.001	<0.001
Serum CEA (ng/ml)	1.8 ± 1.2	70.1 ± 183.6	26.9 ± 106.9	<0.001	<0.001
PE/S CEA	1.0 ± 3.5	21.1 ± 76.2	5.3 ± 12.9	<0.001	<0.001
PE CA15-3 (U/ml)	7.4 ± 10.3	96.5 ± 174.3	30.9 ± 98.9	<0.001	0.080
Serum CA15-3 (U/ml)	8.9 ± 9.2	35.4 ± 77.7	27.6 ± 85.3	<0.001	0.001
PE/S CA15-3	0.9 ± 0.9	3.6 ± 7.3	1.2 ± 2.0	<0.001	0.190
PE CA125 (U/ml)	1,310.3 ± 1,168.2	2,148.2 ± 1,562.3	1,549.2 ± 1,303.9	<0.001	0.091
Serum CA125 (U/ml)	133.6 ± 141.9	258.7 ± 650.5	164.8 ± 342.2	0.033	0.794
PE/S CA125	21.4 ± 60.9	29.7 ± 56.1	22.5 ± 51.0	<0.001	0.586
PE CA19-9 (ng/ml)	9.9 ± 91.9	298.6 ± 616.5	136.6 ± 424.8	<0.001	<0.001
Serum CA19-9 (ng/ml)	12.6 ± 50.8	102.2 ± 331.5	55.1 ± 213.9	<0.001	<0.001
PE/S CA19-9	0.7 ± 4.1	11.6 ± 47.8	5.9 ± 31.8	<0.001	<0.001
PE CYFRA 21-1 (ng/ml)	36.2 ± 49.1	161.3 ± 177.7	89.6 ± 120.7	<0.001	<0.001
Serum CYFRA 21-1 (ng/ml)	2.3 ± 1.9	11.0 ± 27.9	8.4 ± 14.2	<0.001	<0.001
PE/S CYFRA21-1	23.3 ± 38.0	29.6 ± 45.2	21.2 ± 39.4	0.120	0.038
PE NSE (ng/ml)	15.2 ± 39.2	33.1 ± 60.8	16.4 ± 32.0	<0.001	0.912
Serum NSE (ng/ml)	12.9 ± 8.1	20.8 ± 30.0	17.8 ± 16.3	<0.001	<0.001
PE/S NSE	1.3 ± 3.0	1.9 ± 3.5	1.1 ± 2.4	<0.001	0.035

PE, pleural effusion; MPE, malignant pleural effusion; BPE, benign pleural effusion; C/F, current/former; CEA, carcinoembryonic antigen; PE/S, pleural effusion/serum; CA15-3, carbohydrate antigen 15-3; CA125, carbohydrate antigen 125; CA19-9, carbohydrate antigen 19-9; CYFRA21-1, cytokeratin 19 fragment; NSE, neuron-specific enolase.

Data were presented as mean ± standard deviation (SD) or number (percentage). *p* < 0.05 was considered to be statistically significant.

a Comparisons were performed between BPE group and total MPE group using Mann-Whitney U test and Chi-squared (χ^2^) test.

b Comparisons were performed between BPE group and cytology-negative MPE group using Mann-Whitney U test and Chi-squared (χ^2^) test.

### The Diagnostic Performance of Tumor Markers for Total Malignant Pleural Effusion

R Studio software with the OptimalCutpoints package was used to determine the cut-off value of each variable. We defined an AUC greater than 0.75 as an effective tumor marker. The detailed diagnostic reference index of all tumor markers and their corresponding PE/S is presented in Table 3. The cut-offs and AUCs of effective tumor markers were as follows: 3.7 ng/ml [AUC, 0.890 (0.871–0.907)] for PE CEA, 3.6 ng/ml [AUC, 0.834 (0.808–0.859)] for serum CEA, 1.5 [AUC, 0.811 (0.782–0.840)] for PE/S CEA, 9.2 ng/ml [AUC, 0.758 (0.727–0.789)] for PE CA19-9, 59.2 ng/ml [AUC, 0.764 (0.735–0.793)] for PE CYFRA21-1, and 3.0 ng/ml [AUC, 0.852 (0.830–0.874)] for serum CYFRA21-1. Compared to the other five tumor markers, higher diagnostic accuracy of CEA was shown in PE (AUC, 0.890; sensitivity, 74.1%; specificity, 95.5%), serum (AUC, 0.834; sensitivity, 64.4%; specificity, 92.2%), and PE/S (AUC, 0.811; sensitivity, 63.7%; specificity, 94.5%). Besides, PE CEA showed the highest PPV (90.5%), NPV (86.4%), and PLR (16.47), and lowest NLR (0.27) among all variables. With the optimal cut-off value, PE CA15-3 showed the highest specificity (96.9%) among all variables and a higher PLR (16.06), however, the AUC [0.743 (0.712–0.775)] and sensitivity (49.6%) of PE CA15-3 were relatively lower.

**TABLE 3:** Diagnostic performance of six tumor markers in PE, serum and PE/S for total MPE.

Variables	Cut-off	AUC (95%CI)	Sensitivity (%)	Specificity (%)	PPV (%)	NPV (%)	PLR	NLR
PE CEA (ng/ml)	3.7	**0.890 (0.871–0.907)**	74.1	95.5	90.5	86.4	16.47	0.27
Serum CEA (ng/ml)	3.6	**0.834 (0.808–0.859)**	64.4	92.2	82.7	81.7	8.21	0.39
PE/S CEA	1.5	**0.811 (0.782–0.840)**	63.7	94.5	87.0	81.8	11.53	0.38
PE CA15-3 (U/ml)	22.8	0.743 (0.712–0.775)	49.6	96.9	90.3	76.8	16.06	0.52
Serum CA15-3 (U/ml)	13.6	0.711 (0.680–0.742)	47.8	87.3	68.6	74.2	3.76	0.60
PE/S CA15-3	1.5	0.674 (0.640–0.708)	45.8	89.1	70.9	73.9	4.19	0.61
PE CA125 (U/ml)	2056.7	0.662 (0.631–0.694)	42.7	81.4	57.1	71.0	2.29	0.70
Serum CA125 (U/ml)	243.3	0.536 (0.503–0.570)	21.5	86.1	47.3	65.4	1.55	0.91
PE/S CA125	11.3	0.588 (0.556–0.621)	60.2	55.4	43.9	70.5	1.35	0.72
PE CA19-9 (ng/ml)	9.2	**0.758 (0.727–0.789)**	53.3	92.3	80.1	77.3	6.91	0.51
Serum CA19-9 (ng/ml)	25.0	0.653 (0.620–0.686)	31.4	94.0	75.1	70.2	5.20	0.73
PE/S CA19-9	0.8	0.720 (0.688–0.753)	56.2	87.9	73.0	77.6	4.65	0.50
PE CYFRA21-1 (ng/ml)	59.2	**0.764 (0.735–0.793)**	57.3	86.0	70.4	77.6	4.09	0.50
Serum CYFRA21-1 (ng/ml)	3.0	**0.852 (0.830–0.874)**	75.9	79.0	67.8	84.9	3.62	0.31
PE/S CYFRA21-1	53.5	0.527 (0.493–0.560)	16.6	90.7	51.0	65.2	1.79	0.92
PE NSE (ng/ml)	16.5	0.648 (0.616–0.680)	40.7	81.6	56.3	70.3	2.21	0.73
Serum NSE (ng/ml)	12.5	0.681 (0.650–0.711)	66.4	61.1	49.8	75.8	1.70	0.55
PE/S NSE	0.9	0.580 (0.547–0.613)	45.4	67.6	44.9	68.0	1.40	0.81

PE, pleural effusion; PE/S, pleural effusion/serum; CEA, carcinoembryonic antigen; CA15-3, carbohydrate antigen 15-3; CA125, carbohydrate antigen 125; CA19-9, carbohydrate antigen 19-9; CYFRA21-1, cytokeratin 19 fragment; NSE, neuron-specific enolase; AUC, area under the curve; PPV, positive predictive value; NPV, negative predictive value; PLR, positive likelihood ratio; NLR, negative likelihood ratio.

Area under the curve (AUC) was presented as percentage with corresponding 95% confidence intervals (95% CI). The bold values mean the variables with a AUC > 0.75.

### The Diagnostic Performance of Tumor Markers in Combination for Total Malignant Pleural Effusion

**FIGURE 1:**
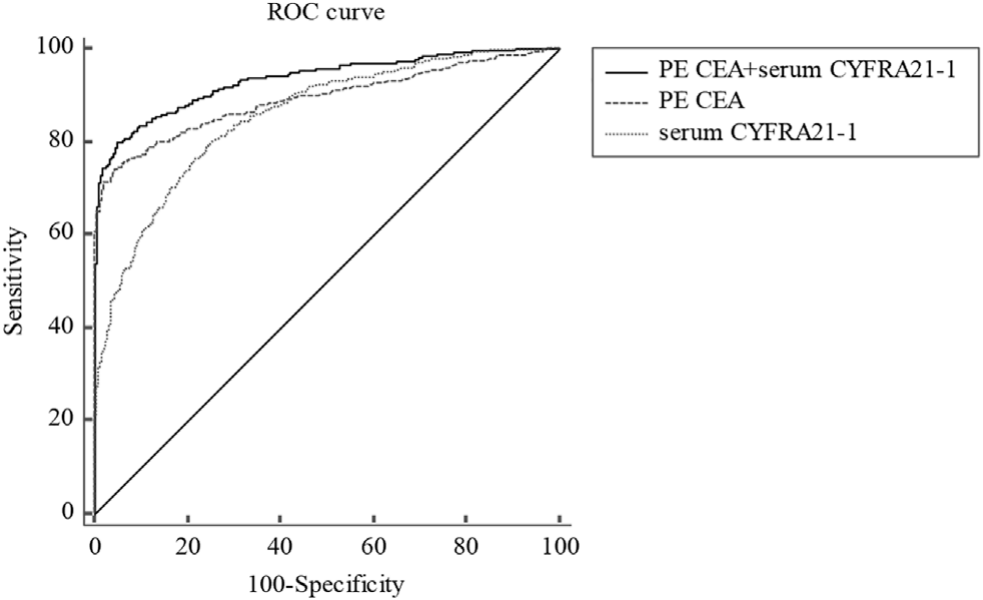
ROC curve for PE CEA, serum CYFRA21-1, and corresponding combinations in distinguishing total MPE from BPE. ROC, receiver operating characteristic; PE, pleural effusion; CEA, carcinoembryonic antigen; CYFRA21-1, cytokeratin 19 fragment; MPE, malignant pleural effusion; BPE, benign pleural effusion.

We also assessed the diagnostic performance of different tumor markers in combination in distinguishing total MPE from BPE. Indicators with AUC greater than 0.75 were used to confirm diagnosis. Since PE CEA had the largest AUC, other indicators were used for joint diagnosis with PE CEA. As shown in Table 4, the combination of PE CEA and serum CEA showed the highest specificity (97.7%), PPV (94.7%), and PLR (30.6) among all combinations, however, the AUC [0.888 (0.869–0.905)], sensitivity (70.8%), and NPV (85.2%) of PE CEA and serum CEA were relatively lower (Table 4). Considering all combinations of indicators, PE CEA and serum CYFRA 21-1 showed the best diagnostic performance with the highest AUC of 0.934 (0.919–0.947) compared with other combinations (Figure 1; Table 4; ). The sensitivity, specificity, PPV, NPV, PLR, and NLR of PE CEA and serum CYFRA 21-1 were 79.9%, 95.7%, 90.5%, 89.0%, 17.35, and 0.21, respectively (Table 4).

**TABLE 4:** The combinations of CEA, CA19-9, and CYFRA21-1 in PE, serum and PE/S for differentiating total MPE from BPE.

Tumor markers	AUC (95% CI)	Sensitivity (%)	Specificity (%)	PPV (%)	NPV (%)	PLR	NLR
PE CEA (ng/ml)	0.890 (0.871–0.907)	70.4	95.5	90.5	86.4	16.47	0.27
PE CEA + PE CA19-9	0.895 (0.877–0.912)	74.8	95.2	90.1	86.7	15.72	0.26
PE CEA + PE CYFRA21-1	0.902 (0.884–0.918)	77.2	92.4	85.5	87.5	10.18	0.25
PE CEA + serum CEA	0.888 (0.869–0.905)	70.8	97.7	94.7	85.2	30.60	0.30
PE CEA + PE/S CEA	0.886 (0.867–0.903)	72.4	96.0	91.3	85.7	18.16	0.29
PE CEA + serum CYFRA21-1	**0.934 (0.919–0.947)**	**79.9**	**95.7**	**90.5**	**89.0**	**17.35**	**0.21**
PE CEA + serum CYFRA21-1+serum CEA	0.932 (0.917–0.946)	81.0	93.2	87.4	89.4	11.89	0.20
PE CEA + serum CYFRA21-1+PE/S CEA	0.933 (0.917–0.946)	79.2	95.1	90.4	88.7	16.22	0.22
PE CEA + serum CYFRA21-1+PE CA19-9	0.934 (0.919–0.948)	80.1	95.0	90.3	89.1	15.98	0.21
PE CEA + serum CYFRA21-1+PE CYFRA21-1	0.933 (0.920–0.946)	81.4	94.1	88.9	89.7	13.77	0.20

PE, pleural effusion; PE/S, pleural effusion/serum; MPE, malignant pleural effusion; AUC, area under the curve; CI, confidence interval; PPV, positive predictive value; NPV, negative predictive value; PLR, positive likelihood ratio; NLR, negative likelihood ratio; CEA, carcinoembryonic antigen; CA19-9, carbohydrate antigen 19-9; CYFRA21-1, cytokeratin 19 fragment. The bold values mean the single tumor marker or combinations of tumor markers with the largest AUC.

### The Diagnostic Performance of Tumor Markers for Cytology-Negative Malignant Pleural Effusion

**FIGURE 2:**
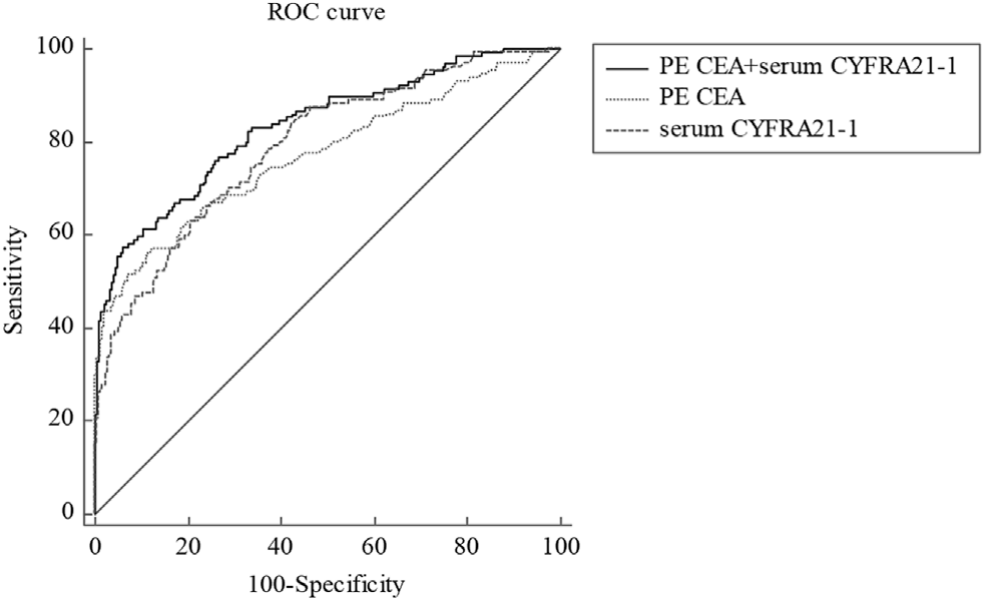
ROC curve for PE CEA, serum CYFRA21-1, and corresponding combinations in distinguishing cytology-negative MPE from BPE. ROC, receiver operating characteristic; PE, pleural effusion; CEA, carcinoembryonic antigen; CYFRA21-1, cytokeratin 19 fragment; MPE, malignant pleural effusion; BPE, benign pleural effusion.

The cut-offs and AUCs of effective tumor markers for diagnosing cytology-negative MPE were as follows: 2.4 ng/ml [AUC, 0.769 (0.740–0.796)] for PE CEA and 3.0 ng/ml (AUC, 0.789 (0.761–0.815)) for serum CYFRA21-1 (Table 5). The combination of serum CYFRA21-1 and PE CEA showed the best diagnostic value in distinguishing cytology-negative MPE from BPE with an AUC of 0.834 (0.808–0.858), higher sensitivity (67.7%), specificity (91.0%), PPV (61.5%), and PLR (9.55) than a single tumor marker (Figure 2; Table 5).

**TABLE 5:** Diagnostic performance of PE CEA and serum CYFRA21-1 for cytology-negative MPE.

Variables	Cut-off	AUC (95%CI)	Sensitivity (%)	Specificity (%)	PPV (%)	NPV (%)	PLR	NLR
PE CEA (ng/ml)	2.4	0.769 (0.740–0.796)	56.2	88.2	44.2	92.3	4.75	0.50
serum CYFRA21-1 (ng/ml)	3.0	0.789 (0.761–0.815)	63.1	79.1	33.5	92.8	3.01	0.47
PE CEA + serum CYFRA21-1		0.834 (0.808–0.858)	67.7	91.0	61.5	93.0	9.55	0.45

PE, pleural effusion; CEA, carcinoembryonic antigen; CYFRA21-1, cytokeratin 19 fragment; AUC, area under the curve; PPV, positive predictive value; NPV, negative predictive value; PLR, positive likelihood ratio; NLR, negative likelihood ratio.

Area under the curve (AUC) was presented as percentage with corresponding 95% confidence intervals (95% CI).

## Discussion

In our study, we compared the levels of PE, serum, and PE/S of CEA, CA15-3, CA125, CA19-9, CYFRA 21-1, and NSE to determine the best diagnostic tumor marker for MPE. Our results indicated that PE CEA was the best indicator to diagnose MPE with a cut-off value of 3.7 ng/ml (AUC, 0.890 (0.871–0.907)). PE CEA at 3.7 ng/ml showed higher sensitivity, specificity, PPV, and NPV for MPE when compared to another single indicator in PE, serum, and PE/S. Our study is in accordance with other previous studies [8, 9]. Huang et al. indicated that PE CEA provided better diagnostic performance in discriminating lung adenocarcinoma-associated MPE (LAC-MPE) from BPE than PE HER2/neu (human epidermal growth factor receptor 2/neutrophil) and PE CYFRA21-1 [10]. Similarly, another study assessing the best tumor marker for differentiating LAC-MPE from BPE obtained the same results about PE CEA [11]. Besides, Feng et al. showed that PE CEA exhibited the best diagnostic performance for differentiating LAC-MPE from BPE with an AUC of 0.95 as well as high sensitivity (87.65%) and specificity (93.75%) compared with PE CYFRA21-1 and CA19-9 [12]. Recently, a similar study from China also showed that PE CEA was the best effective indicator for diagnosing lung cancer-associated MPE among the five tumor markers (CEA, CYFRA 21-1, SCC-Ag, CA125, and NSE) at a cut-off value of 5.23 ng/ml [13]. Results showed the cut-off values, sensitivities, and specificities of PE CEA for diagnosis of MPE were widely inconsistent, probably due to different detection methods, sample sizes, and types of included cancers. Besides, several studies showed that PE CEA had dramatically low sensitivities, probably due to cases of mesothelioma and hematological tumor origin [14-17]. CEA was not increased when PE was derived from mesothelioma, lymphoma, and leukemia. In addition, studies involving only lung cancer have higher sensitivity and AUC than studies involving various cancers, which might also contribute to the inconsistencies of PE CEA [7, 15, 18-22].

CEA, a glycoprotein component of the glycocalyx, is the earliest fetal embryo antigen involved in cell adhesion. It is usually generated during fetal development, but does not appear in the peripheral blood of healthy individuals [23, 24]. Currently, serum CEA is the most used biomarker for the diagnosis and prognosis of several malignant diseases, such as lung cancer and colorectal cancer. However, PE CEA has been proven to be a more effective indicator for the diagnosis of MPE than serum CEA in previous studies [9-13, 19]. The potential mechanism was that the tumor cells metastasize to the pleural cavity by the direct invasion of the pleura or blood [25]. Therefore, the invaded tumor cells directly secreted tumor markers into the pleural cavity or the blood, which were diluted [26]. Moreover, tumor cells might block lymphatic drainage, reducing tumor markers in the blood. Therefore, tumor markers were concentrated in the pleural cavity [27].

Though PE CEA showed a good diagnostic value in distinguishing MPE from BPE, a combination of multiple tumor markers to diagnose MPE might be more valuable in clinical practice. Several studies have recommended that the diagnostic value of combinations of two or more tumor markers was greater than any single tumor marker for diagnosing MPE [8, 9, 12]. In a meta-analysis, the authors explored the diagnostic accuracies of combinations of several tumor markers (CEA, CA125, CA15-3, CA19-9, and CYFRA 21-1) for MPE[9]. The results showed that the combination of PE CEA plus CA 15-3 and PE CEA plus CA 19-9 highly indicated MPE, however, the sensitivity of these combinations was quite poor [9]. In addition, the aforementioned three studies suggested that the combination of CEA and CYFRA 21-1 exhibited a higher diagnostic performance than any single index and other combinations [10, 12, 28]. Our results were in accordance with previously reported studies [10, 12, 28]. CYFRA21-1, a fragment of cytokeratin 19 (CK19), is a common marker for epithelial malignant origin, which reflects ongoing cell activity. Increased protease activity of caspase 3 in neoplastic-transformed epithelial cells degrades CK19, which releases the fragment into the peripheral circulation. Hence, increased CYFRA 21-1 is a tumor marker reflecting the occurrence of epithelial neoplasms [29]. In the present study, the AUCs of PE CYFRA21-1 and serum CYFRA21-1 were 0.764 and 0.852, respectively. However, the sensitivity, specificity, PPV, and PLR were relatively poor.

Therefore, we used multiple indicators for joint diagnosis of MPE. In our study, the combination of PE CEA and serum CYFRA21-1 showed the highest AUC [0.934, 95% CI (0.919–0.947)] with 79.9% sensitivity and 95.7% specificity when compared with any other combinations of indicators. Besides, the PPV of the combination was 90.5%, which indicated the likelihood of developing MPE in the patients. PLR and NLR integrated advantages of sensitivity, specificity, PPV, and NPV for disease diagnosis, which were not affected by the incidence of disease. Therefore, they were relatively independent, clinically significant indexes of diagnostic test evaluation. When PLR > 10 or NLR < 0.1, the likelihood of diagnosis or exclusion of disease was significantly increased. The PLR of PE CEA and serum CYFRA21-1 in diagnosing MPE was 17.35, indicating the significantly increased diagnostic accuracy of MPE.

The diagnosis of MPE is currently based on finding tumor cells in PE or tissue. However, whether tumor cells are detected in PE or not depends on the pathologist’s experience, tumor histologic type, and degree of pleural invasiveness [30]. Although thoracoscopy can diagnose about 90% of PE cases, this method is not always feasible, especially in patients with advanced disease and unstable clinical conditions. Therefore, the limited sensitivity of PE cytology has forced us to seek new auxiliary diagnostic methods to improve the reliability of diagnoses, especially in cytology-negative cases. Hsieh et al. evaluated the diagnostic value of HER2/neu, CYFRA21-1, and CEA to distinguish LAC-associated cytologically negative PE (LAC-CNPEs) from BPEs [28], but the sensitivities of the three markers were poor [28]. However, the combination of CEA and CYFRA21-1 increased the sensitivity to 66.7%. Another study conducted by Antonangelo et al. indicated that PE CA125 might be used to distinguish cytology-negative MPE from BPE [7]. Therefore, we evaluated the diagnostic value of tumor markers in distinguishing cytology-negative MPE from BPE. Our results indicated that the combination of PE CEA and serum CYFRA21-1 showed a better diagnostic performance in distinguishing cytology-negative MPE from BPE with an AUC of 0.834 than single PE CEA or serum CYFRA21-1. The sensitivity, specificity, PPV, and PLR were 67.7%, 91.0%, 61.5%, and 9.55, respectively. Previously, we also developed and validated a scoring system based on a nomogram for distinguishing MPE and BPE, which performed well for differentiating lung cancer and tuberculosis [31]. However, the diagnosis of MPE was according to the presence of malignant cells in PE cytology in our previous study [31]. At present, many clinicians still regard positive cytology as the gold standard for MPE diagnosis, but the accuracy rate of this method is only about 60% [3, 7]. The medical burden and the poor quality of life for patients caused by missed diagnosis cannot be ignored. Therefore, in the present study, we not only compared these common tumor markers between total MPE and BPE, but more importantly, we also explored the diagnostic value of these tumor indicators in cytology-negative MPE, which might be beneficial to improve the diagnostic accuracy for patients with MPE. The patients used in our two articles were overlapped. The reasons for the overlapping patient cohort were mainly due to the differences in the analyzed variables, the statistical time, and whether mesothelioma and hematologic tumors were excluded or not. In general, our results might be useful in the complementary diagnosis of MPE when cytology is suspected or negative, avoiding more invasive procedures in clinical practice. Besides, the detection of two tumor markers is convenient and can be detected in most hospitals, which is beneficial for early clinical decision making.

To the best of our knowledge, the sample size of our study was the largest, and various types of tumors were included in our study. Besides, the sample sizes of previously reported studies included no more than 300 individuals, and many studies were about lung cancer-associated PE, which may lead to statistical bias. Moreover, the tumor markers in PE, serum, and their corresponding ratio in our study were comprehensively investigated in distinguishing MPE from BPE. We also used a combination of two or more indicators to diagnose MPE, especially in cytology-negative MPE. Therefore, our study will provide an early and accurate reference for the auxiliary diagnosis of MPE, which is beneficial to early treatment and prognosis.

However, our study has several limitations. First, the study was a single-center retrospective study. More prospective and multicenter studies with different populations should be carried out to validate our findings. Second, we did not compare the diagnostic value of tumor markers in discriminating LAC-MPE from other causes of MPE, though LAC-MPE accounted for the majority of MPE sources in clinical practice. Therefore, further studies should focus on discriminating LAC-MPE from other causes of MPE. Third, the diagnostic sensitivity for MPE in our study was not high. We were unable to combine the markers with thoracoscopy or imaging examination for MPE diagnosis due to unavailable data.

In summary, PE CEA at a cut-off value of 3.7 ng/ml showed the best diagnostic performance in distinguishing MPE from BPE. PE CEA and serum CYFRA21-1 were effective diagnostic tumor markers in distinguishing cytology-negative MPE from BPE. Combinations of PE CEA and serum CYFRA21-1 could increase the diagnostic accuracy in distinguishing MPE from BPE and cytology-negative MPE from BPE.

## Data Availability

The original contributions presented in the study are included in the article/Supplementary Material, further inquiries can be directed to the corresponding author.
